# Multicomponent Network Formation in Selective Layer of Composite Membrane for CO_2_ Separation

**DOI:** 10.3390/membranes11030174

**Published:** 2021-02-28

**Authors:** Jelena Lillepärg, Evgeni Sperling, Marit Blanke, Martin Held, Sergey Shishatskiy

**Affiliations:** Helmholtz-Zentrum Geesthacht, Institute of Polymer Research, Max-Planck-Str. 1, 21502 Geesthacht, Germany; evgeni.sperling@hzg.de (E.S.); marit.blanke@gmx.de (M.B.); martin.held@hzg.de (M.H.); sergey.shishatskiy@hzg.de (S.S.)

**Keywords:** network formation, thin film composite membrane, gas separation, PolyActive^TM^

## Abstract

As a promising material for CO_2_/N_2_ separation, PolyActive^TM^ can be used as a separation layer in thin-film composite membranes (TFCM). Prior studies focused on the modification of PolyActive^TM^ using low-molecular-weight additives. In this study, the effect of chemical crosslinking of reactive end-groups containing additives, forming networks within selective layers of the TFCM, has been studied. In order to understand the influence of a network embedded into a polymer matrix on the properties of the resulting materials, various characterization methods, including Fourier transform infrared spectroscopy (FTIR), gas transport measurements, differential scanning calorimetry (DSC) and atomic force microscopy (AFM), were used. The characterization of the resulting membrane regarding individual gas permeances by an in-house built “pressure increase” facility revealed a twofold increase in CO_2_ permeance, with insignificant losses in CO_2_/N_2_ selectivity.

## 1. Introduction

Membrane technology is a promising technology for the separation of greenhouse gases such as CO_2_ from industrial and energy generation exhaust gases. CO_2_ separation from the flue gas in post combustion processes, biogas and natural gas purification are just a few applications where membranes are successfully competing with absorption- and adsorption-based processes. Low membrane prices due to the low consumption of the selective layer material, the ease of assembly in modules and the ease of scaling up are some of the advantages of separation processes based on polymeric membranes and especially TFCM [1,2,3,4,5,6].

Much of the earlier work on membrane design was performed to find the best relationship between the permeability and selectivity of the selective layer material. The PolyActive^TM^ block copolymer became a very promising material for CO_2_/N_2_ separation due to its high content of soft poly(ethylene) oxide (PEO) blocks, having a high affinity towards CO_2_. The mechanical resilience of PolyActive^TM^, under conditions where separation is still feasible, is maintained through the polybutylene terephthalate (PBT) crystalline domains. Detailed studies of the effect of the PEO‒PBT ratio on the gas transport properties of TFCM are described elsewhere [7,8,9,10].

The incorporation of PEO-containing additives into the polymer matrix in order to increase CO_2_ permeability has been widely investigated in the last few decades. An increase in permeability compared to the initial polymer with minimum selectivity losses makes it possible to reduce the membrane area and, accordingly, the investment costs needed. However, there are some drawbacks since additives with a high PEO molecular weight can strongly increase the crystallization temperature of the material of the separation layer and shift the operating conditions of the membrane to an inappropriate temperature. Furthermore, the most effective low-molecular-weight (LMW) additives leach out from the polymer matrix [6,11,12,13,14].

In order to prevent the leakage of LMW additives from polymers, substances with reactive end groups that can be crosslinked directly in the polymer can be used. Similar systems with a network formation of LMW compounds have been widely studied in the field of gelators [15,16,17].

The interpenetration of thermally crosslinked networks using amine-containing poly(ethylene glycol)s (PEGs) was thoroughly studied for thin-film composite membranes with Pebax^®^ separation layers. An increase in CO_2_ permeability, accompanied by high stability of CO_2_/N_2_ selectivity, was observed for PEG methyl ether acrylate membranes. Increases in both permeability and CO_2_/N_2_ selectivity were observed for the PEG–Jeffamine^®^ crosslinked system [18,19,20].

In the present study, we investigate the influence of poly(ethylene/-propylene glycol)-containing interpenetrating networks generated by the reaction of amine-containing Jeffamine^®^-PEGs and epoxy glycidyl-containing PEGs within the PolyActive™ selective layer of TFCM with a view to using the obtained membrane for CO_2_/N_2_ separation in postcombustion carbon capture systems. The crosslinking was carried out via a widely used thermally activated ring-opening reaction between glycidyl- and amine-terminated LMW PEG-based additives [15,21,22].

TFCMs containing PolyActive^TM^ selective layers were prepared by coating from solution. The compatibility of polymer with LMW compounds was investigated by Hansen solubility parameters (HSP) calculations, gas transport properties investigation and FTIR. The morphology of the obtained membranes was assessed by atomic force microscopy (AFM) and scanning electron microscopy (SEM).

## 2. Materials and Methods 

### 2.1. Materials and Preparation of Samples

PolyActive^TM^ 1500PEGT77PBT23 (further P1500) was purchased from PolyVation BV (Groningen, The Netherlands) in pellet form. Poly(propylene glycol) diglycidyl ethers PPG380 and PPG640, poly(ethylene glycol) diglycidyl ether (PEG526), trimethylolpropane triglycidyl ether (TPT302), poly(ether diamine)s ED series or Jeffamine^®^ O,O’-Bis (2-aminopropyl) polypropylene glycol-block-polyethylene glycol-block-polypropylene glycol JED600, JED 900 and JED 2003 were obtained from Sigma-Aldrich Chemie GmbH (Hamburg, Germany). Jeffamine^®^ trimethylolpropane poly(oxypropylene)triamine (JT403) was purchased from Huntsman Corporation (Everberg, Belgium). An overview of the components used in the experiments, their basic properties and short names is presented in Table 1. Chemical structures are provided in Table S1.

Tetrahydrofuran (THF) was purchased from Th. Geyer GmbH & Co. KG (Renningen, Germany). All materials were used as received without any further purification.

P1500 films with thickness in the range 60–100 µm were prepared by casting a 3 wt % polymer solution in THF at 25 °C. The solution was obtained under reflux conditions with stirring for 3 h. For the preparation of P1500 films blended with amine- and epoxy-containing additives, the polymer solution was cooled to 25 °C and the required amounts of the aforementioned materials were added. The solution was stirred for 20 min at room temperature and then poured through a cotton filter into an aluminum cylinder with a polished bottom edge placed on a leveled Teflon^TM^-coated glass plate [14]. The formed beaker containing the polymer solution was covered with a glass lid equipped with connections for slow nitrogen flow in and out. This arrangement allowed for slow THF evaporation at 25 °C. As a result, within 48 h, an isotropic, mostly solvent-free film was formed. 

All prepared films were further degassed for 2 h in a vacuum oven at 90 °C to remove any residual volatile compounds. In the case of blends, the crosslinking reaction of PEGs with reactive end-groups took place during the film’s exposure to 90 °C temperature, according to a previously described procedure [18].

Thin-film composite membranes (TFCM) were prepared from a 1.5% polymer solution in THF on a multilayer TFCM consisting of a polydimethylsiloxane gutter layer deposited on a porous poly(acrylonitrile) layer [23], as developed in Helmholtz–Zentrum Geesthacht (HZG). The gutter layer provides a smooth surface for the deposition of the separation layer and improved drainage of the permeate into the porous support structure. All blended TFCMs were thermally treated in a vacuum oven at 90 °C for 2 h. The morphology of the prepared TFCMs is presented in Figure S1.

### 2.2. Sample Characterization

Hansen solubility parameters (HSP) *δ* and molar volumes $V_{p}$ of the components used for blends were estimated according to the data collected by Fedors and presented by Hoftyzer and van Krevelen [24]. The total solubility parameter can be estimated as the sum of the squares of the Hansen components:

(1) $$\delta_{t}={\left(\delta_{d}^{2}+\delta_{p}^{2}+\delta_{h}^{2}\right)}^{1/2},$$

where *d* refers to dispersion bonding, *p* to polar bonding and *h* to hydrogen bonding.

Carbon dioxide is known as a nonpolar but polarizable gas (e.g., static dipole polarizability for CO_2_ is 16.92 a.u.; for O_2_ is 10.54 a.u.; for N_2_ is 11.54 a.u. [25]) that interacts with most solvents due to dispersion forces [26]. The solubility parameter distance *Ra* between component *k* and CO_2_ as solvent *s* was calculated by the following equation:

(2) $${\left(Ra\right)}^{2}=4{\left(\delta_{dk}-\delta_{ds}\right)}^{2}+{\left(\delta_{pk}-\delta_{ps}\right)}^{2}+{\left(\delta_{hk}-\delta_{hs}\right)}^{2}.$$

The molar volume of the selected components was calculated using the HyperChem^®^ molecular modeling software. The energy minimization algorithm allows for geometry optimization of the designed molecule and estimation of the van der Waals surface and volume.

The estimation of the solubility parameters for blended polymers was performed using the following equation [27]:

(3) $$\delta =w_{1}\cdot \delta_{1}+w_{2}\cdot \delta_{2},$$

where *w*_1_ and *w*_2_ are the weight fractions of the components.

Differential scanning calorimetry (DSC) experiments were performed for P1500, Jeffamine^®^s and glycidyl-containing additives in a DSC1 (Starsystem) from Mettler Toledo (Ockerweg, Germany), at a scan rate of 10 K/min, using nitrogen as purge gas stream. Heating and cooling scans were performed by initially heating the sample up to 200 °C and holding it at that temperature for 5 min in order to erase the previous history. Afterwards, the samples were cooled down to –30 °C and a second heating scan up to 200 °C and a second cooling scan down to 30 °C were done. The presented values obtained from the DSC experiment correspond to the first cooling and the second heating scans.

For detecting the dynamic viscosity change of a mixture of P1500 and blends with reactive end-groups, an integrated vibrational viscometer MIVI 5000 (Sofraser S.A., Villemandeur, France) was used. The measurement was performed in a sealed thermally controlled sample vessel at a constant stirring speed of 1000 min^−1^ with a magnetic drive cyclone 075 (Büchi AG, Uster, Switzerland). The viscosity range of the sensor was 0.01–100) mPa·s and the viscosity was recorded every 10 s. The dynamic viscosity measurement accuracy is ±2% of its range [28].

The thickness of the prepared polymeric films was measured with a DELTASCOPE^®^ FMP10 digital micrometer (Fischer, Sindelfingen, Germany), with an accuracy of up to ±4% of 20 measurements for each sample.

The density measurements of polymeric films were performed by the Archimedes principle using an Excellence Plus Mettler Toledo analytical balance (Gießen, Germany). In order to prevent any influence of a liquid sorption on the density values, FLUORINERT^TM^ FC-77 from 3M (St. Paul, MN, USA) was used as the auxiliary liquid [29].

The permeability coefficients *P* (Barrer, 1 Barrer = 1 × 10^−10^ cm^3^ (STP) cm cm^−2^ s^−1^ cmHg^−1^) and diffusion coefficients *D* (cm^2^ s^−1^) of individual gases for the prepared films were obtained in an in-house developed “time-lag” facility utilizing the constant volume/variable pressure principle. The solubility coefficient was estimated from a relationship between permeability and diffusion coefficients:

(4) $$S=\frac{P}{D}.$$

Gas transport properties were measured at least three times for each gas and each sample at feed pressures of 60, 45, or 30 kPa and permeate pressure of 0 Pa to 100 Pa.

Gas permeances *L_i_* for an individual gas *i* for TFCMs were measured using the in-house developed “pressure increase” facility, utilizing the same measurement principle as in the “time-lag” facility but with the software optimized for characterization of membranes with a negligible time lag due to an extremely thin selective layer, as described elsewhere [23,30]. TFCM samples with a selective layer of P1500 and various blends were investigated to determine the gas transport properties in the temperature range 20–80 °C at feed pressures lower than 60 kPa and permeate pressure change from 10 Pa to 130 Pa. The permeance was determined as the average of at least 10 measurements within each analysis. The measurement uncertainty for “time lag” and “pressure increase” measurements was calculated according to a method described elsewhere [30], which considers the influence of the errors in the measurements of each sensor.

Ideal selectivity for gases *x* and *y* was determined as a ratio of permeances:

(5) $$\alpha \left(\frac{x}{y}\right)=\frac{L_{x}}{L_{y}}.$$

High-pressure experiments were conducted with feed pressures up to 3.5 MPa at a constant temperature of 30 °C using the simple experimental facility and utilizing constant pressure/variable volume principle described elsewhere [10]. The permeance was calculated without taking into account the real gas behavior of CO_2_.

To determine chemical changes in the separation layer of TFCM, Fourier transform infrared (FTIR) spectra were recorded in attenuated total reflectance (ATR) mode on a Bruker ALPHA FT-IR spectrometer (Ettlingen, Germany) equipped with a germanium crystal. The transmittance measurements were done at room temperature in a spectral range from 550 cm^−1^ to 4000 cm^−1^ with a resolution of 4 cm^−1^ and an average of 64 scans. The gutter layer’s TFCM spectrum was used as a background for measurements with the ATR unit.

The P1500 membranes were investigated as produced (1.5 wt % solid content in THF solution, 30 wt % additive) by AFM. FTIR and AFM were studied after investigations of the gas transport properties over a wide temperature range. The AFM images of membrane surfaces were obtained by a Bruker MultiMode 8 (Karlsruhe, Germany), in Peak-Force (PF) qualitative nanomechanical mapping (QNM) mode at room temperature (22–26 °C). ScanAsyst-Air probes (tip radius 2 nm, spring constant 0.4 N/m) were applied for these measurements. The PF QNM mode enables the mapping of such material characteristics as elasticity and adhesion via the fitting of force‒distance curves, along with the heightmap. The scan parameters (PF set point and sync distance QNM) were adjusted in order to obtain the maximum elasticity values that roughly match the Young’s modulus of the PBT block (approximately 3 GPa) to obtain semiquantitative QNM maps.

In order to quantify the size of the stiffer domains, we use the full width at half maximum (FWHM), which describes the width of a stiffer phase’s domain at half of the global maximum in the log Derjaguin−Muller−Toporov (DMT) image. For each of the three 3-µm scans per sample, two diagonal cross sections of the log DMT images were used. The obtained values were averaged over all three images per sample to find the mean domain size. The quantification of the stiffer phase’s proportion, i.e., its ratio to the soft phase, results from normalizing the frequency in the histogram of the log DMT images to the soft phase peak. The stiff/soft ratio was averaged over the three scans to obtain a mean value per sample.

SEM images of bulk membranes and TFCMs were obtained by Leo 1550 VP (Zeiss, Oberkochen, Germany). Prior to measurements, the samples were coated with 1 nm Pt by the sputter coater BAL-TEC MED 020, (BAL-TEC, now part of Leica Microsystems, Wetzlar, Germany), to avoid charging effects during the scanning.

## 3. Results and Discussion

### 3.1. Parameters of Individual Compounds

The values for each of the three HSP components, the total solubility parameter and the solubility distance based on room temperature solubility are listed in Table 2. Theoretical solubility parameters for P1500 were estimated as stated above by the group contribution method reported elsewhere [24]. Therefore, in Table 2 the solubility parameters are given for the individual soft and hard segments of P1500. The solubility parameter of CO_2_ has been reported by several authors and ranges from 6.77 MPa^1/2^ by Sebastian to 17.85 MPa^1/2^ by Sistla [26,31,32,33]; hence, the values show large variations. Since CO_2_ has HSP values of ***δ_d_*** = 15.6 MPa^1/2^, ***δ_p_*** = 5.2 MPa^1/2^ and ***δ_h_*** = 5.8 MPa^1/2^, the resulting ***δ_t_*** became 17.4 MPa^1/2^ at 25 °C and a CO_2_ pressure of 1 atm [34]. The best additives should fulfill the criteria 15 MPa^1/2^ < ***δ_t_*** < 20 MPa^1/2^, which is the case for all the compounds listed in Table 2.

As Table 2 shows, CO_2_ can interact better with the soft PEGT segment of P1500. The PBT block at the conditions of the gas transport experiments is in the semicrystalline state and is considered as impermeable barrier for penetrants, a fact reflected in the total solubility and solubility distance parameters, while CO_2_ and other gases’ transport is performed through the rubbery PEO blocks [35]. All materials chosen for blending with P1500 are theoretically able to increase the CO_2_ solubility since the CO_2_ solubility “distance” *Ra* is less than the *Ra* of the soft segment PEGT1500.

The formation of crystallites in the polymer phase that is responsible for gas transport negatively influences the gas transport properties of the polymer. Table 3 presents some thermal properties of materials under investigation. Melting temperatures (*T_m_*) and crystallization temperatures (*T_c_*) for materials with three amino and glycidyl groups were not identified on curves of the DSC investigation. PPGs are liquids; an undefined melting point indicates the atactic structure and the lower crystallinity [36]. The *T_m_* for diamine compounds increases with increasing molecular weight. DSC measurements for selected TFCM with network formation can be found at Table S3.

Data obtained in previous studies on Jeffamine^®^ and LMW PEGs used as additives indicated that the *T_g_* drift for blends is not bigger than ±3 °C. For the blended P1500, a shift in the melting point *T_m_* was observed for different blends, but it was not related to the amount of additive. The similarity of the chemical structure of the PEG-containing additive to the structure of the soft PEO segment of P1500 promotes a stronger change in crystallization temperatures. For samples containing additives, the transition from the crystalline to the molten state can be traced by the change in permeation properties using time lag and pressure increase equipment. A comparison of samples of thick films and TFCM can be difficult due to the ordinal difference in the thickness of the film in which the network is formed. Moreover, long-chain crystallites in TFCM can form separate domains in the separation layer and reduce the expected effect of transport properties’ enhancement [11,18,37,38].

**Figure 1:**
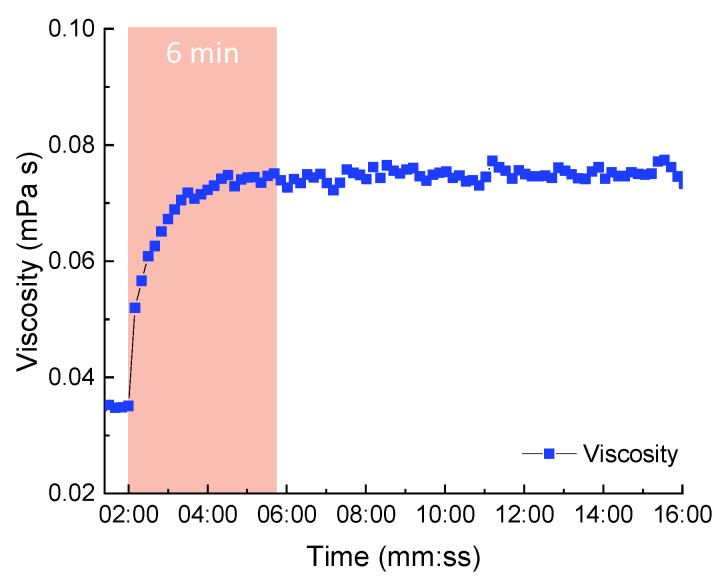
Changes in the viscosity of 5 wt % Jeffamine^®^ JED600 and PEG-glycidyl PEG526 and 5 wt % P1500 in THF and ethyl lactate (1:3) at 40 °C.

Dynamic viscosity measurements were carried out for the 5 wt % solutions of P1500 in a mixture of THF and ethyl lactate at a 1:3 ratio with stirring at 40 °C. After stabilization of the measured viscosity values of the P1500 solution, additives JED600 and PEG526 with two reactive end-groups were added. The concentration of both P1500 and additives in the solution reached 10 wt %. During the measurement, a statistically significant increase of solution viscosity was observed from 0.035 mPa s to 0.075 mPa s within 6 min (Figure 1).

This result suggests that the reaction of glycidyl and amino end-groups will occur at room temperature within just a few minutes. Since this device requires a large amount of material and the reaction proceeds rapidly in a rotational viscometer at an elevated temperature, we do not present data with other combinations of additives.

A rapid reaction with a sufficient amount of additives can occur in the solid polymer matrix, similar to the crosslinking reactions studied for substances in solution. To confirm this, a number of thick film samples with blends were prepared and tested. 

### 3.2. Network Formation within Thick Films

**Figure 2:**
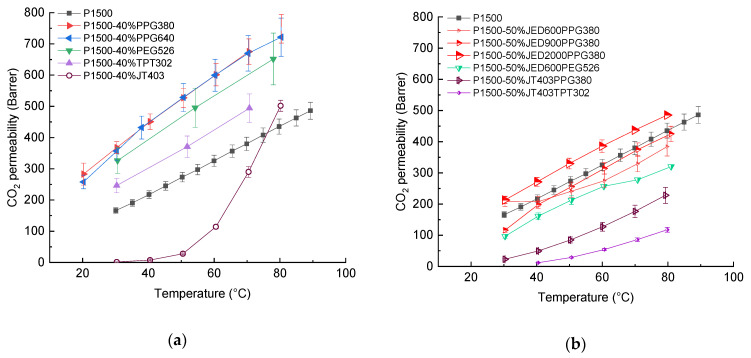
CO_2_ permeabilities of blended thick films of P1500 (**a**) thick films with nonreacted additives; (**b**) thick films with network formation through end-group reaction. Error bars indicate the measurement uncertainty with 95% level of confidence including standard deviation of three specimens of each measurement point.

Preparation of thick P1500 films blended with the compounds listed in Table 1 was successful for all compounds with glycidyl end-groups and for one compound with three amino end-groups. The prepared films were transparent and had thicknesses in the range of 60–100 µm. For both PPG380 and PPG640, a 100% increase in CO_2_ permeability was observed compared to the pristine P1500 (Figure 2a). The PPG chain length did not show any influence on the CO_2_ permeability in the temperature range from 30 °C to 80 °C. The SEM images of the upper surface of thick films exposed to the atmosphere during film preparation are shown in Figure S2. Morphological features observed on the surface of thick films are related to the partial salting out of low molecular compounds during film preparation by slow solvent evaporation.

The significant increase in CO_2_ permeability above 50 °C for the P1500-40%JT403 sample reflects the shift of the melting point for this polymer/additive composition. The compositions containing additives of the Jeffamine^®^ ED series became very sticky and difficult to separate, even from the Teflon^®^ surface. This effect resulted in nonuniform thicknesses of the film, posing a significant challenge to the proper determination of permeability, diffusion and consequently, solubility coefficients.

Samples consisting of P1500 blended with one additive tend to lose permeability in the range of 5–50%, due to the additive leaching out during repeated experiments for gas transport properties determination. Similar results were observed for all studied blends of P1500 with a single LMW compounds. More information was given in our previous study, where leaching was found for all LMW compounds with a molecular mass less than 500 g/mol [14].

Table 4 provides the permeability coefficients for selected gases at 30 °C for the samples shown in Figure 2. As mentioned earlier, a twofold increase in CO_2_ permeability was observed for both PPG380 and PPG640 (Figure 2a, Table 4). This is due to an increase in both solubility and diffusion coefficients. N_2_ permeability increases by 2.5-fold, which results in a slight decrease in CO_2_/N_2_ selectivity. High CO_2_/N_2_ selectivity is also provided by the additive PEG526 (Figure 2) with two glycidyl end groups, in contrast to the component with three end groups, as presented in Table 4. It should be noted that the CO_2_ solubility increases in accordance with changes in calculated HSP *δ_t_* and *Ra* in Table 2. The low CO_2_ diffusion coefficient for TPT302 leads to a low permeability coefficient and can indicate the presence of crystallites in the blend at a given temperature.

The second set of thick P1500 films was prepared as described above with two types of reactive additives. The reaction of amino and glycidyl end-groups should proceed during the heating of solvent-free samples. Based on the results of the experiments with single additive blends and the estimation of solubility parameters, PPG380 was chosen to react with the Jeffamine^®^ EDs. Samples containing JED show a clear increase in CO_2_ permeability, correlating with the JED chain length (Figure 2b). The measured solubility coefficients for the PPG containing Jeffamine^®^ ED series are in accordance with the calculated values of HSP for polymer systems presented in Table 5. In contrast, the addition of a PEG-containing additive and its combination with the shortest JED should have an effect on the overall solubility. As can be seen from Table 4, the CO_2_ solubility of the 50%JED600PEG526 blend was the highest of the considered compositions. Unfortunately, the low diffusion coefficient is responsible for a lower permeability of JED600PEG526 (Figure 2b and Table 4). For the compositions 50%JED600PPG380, 50%JED900PPG380 and 50%JT403TPT302, no data are available at 30 °C for O_2_, N_2_ and CH_4_ due to insufficient film stability. As the ***δ_t_*** and *Ra* values demonstrate for 50%JT403PPG380 (Table 5), the advantaged CO_2_ affinity reflects the high value measured for CO_2_ solubility (Table 4), while the diffusion coefficient is low. CO_2_ permeability measurements for 50%JT403TPT302 were possible at 40 °C and above and for other gases starting at 50 °C. 

Since an unpredictable distribution of additives occurs in the matrix of a thick film, the formation of inhomogeneity is possible in the polymer matrix during the reaction of additives. Thus, there is a mismatch between the calculated HSP for the mixture of components (Table 5) and the solubility coefficient obtained experimentally (Table 4).

### 3.3. Network Formation in TFCM

**Figure 3:**
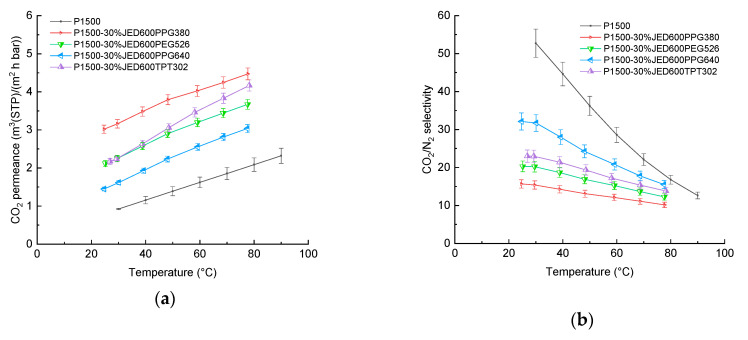
Temperature dependence of (**a**) permeance and (**b**) selectivity of TFCMs with a selective layer of P1500 blended with Jeffamine^®^ having two reactive amino groups and components having two and three glycidyl reactive groups. Error bars indicate the measurement uncertainty with 95% level of confidence, including standard deviation of 10 specimens of each measurement point.

Network formation directly in the separation layer of a TFCM can be influenced by their relatively low thicknesses of about 200 to 400 nm (Table S2) and membrane formation conditions being governed by the fast solvent evaporation [39]. The sample preparation method described above was applied to prevent an early reaction of additives in the casting solution. SEM images, provided in Figure S3, depict the surface morphology of the selective layer. As seen in Figure 3a, the CO_2_ permeance increases in the presence of JED with two reactive amine groups that reacted with additives having two or three glycidyl groups. At the same time, the CO_2_/N_2_ selectivity was found to be significantly lower than that of the unmodified P1500 (Figure 3b). For all compositions, a jump in gas transport properties was observed around 40 °C, which is connected to the melting point shift (Table S2).

**Figure 4:**
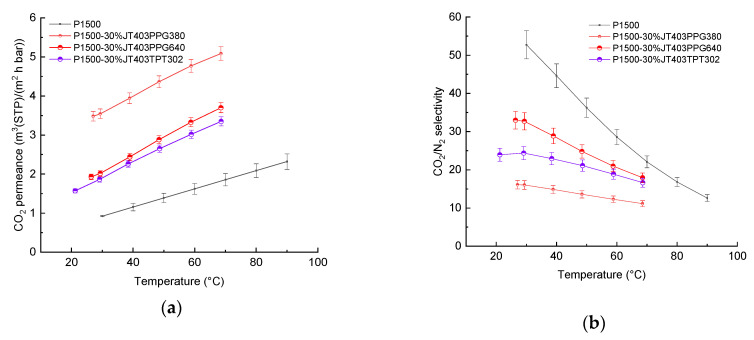
Temperature dependence of (**a**) permeance and (**b**) selectivity for TFCM with a selective layer of P1500 blended with Jeffamine^®^ having three reactive groups and components having two and three glycidyl reactive groups. Error bars indicate the measurement uncertainty with a 95% level of confidence, including the standard deviation of 10 specimens of each measurement point.

Figure 4 shows the results of pressure increase measurements of membrane permeances and ideal selectivities in dependence on temperature for compositions based on Jeffamine^®^ with three end groups that reacted either with PPG with different molecular weights or TPT. It is interesting to note that the increase in permeances for the composition with the smaller molecular weight PPG380 is greater than for compositions with the heaviest component PPG640 and that the values are similar to the results for compositions with JED600. In contrast to the samples studied in our previous work, the samples of this work, when investigated according to the same protocol, give a difference in properties of 0.6–3.5%. For the composition JED600PPG380, the difference is significantly higher and amounts to 9% at 30 °C, which can be explained by the partial salting out of the LMW component [40].

**Figure 5:**
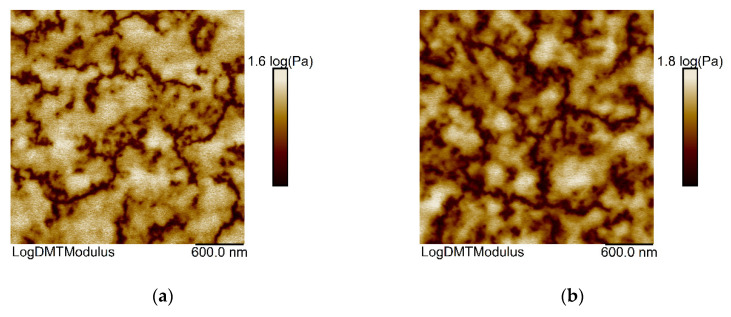

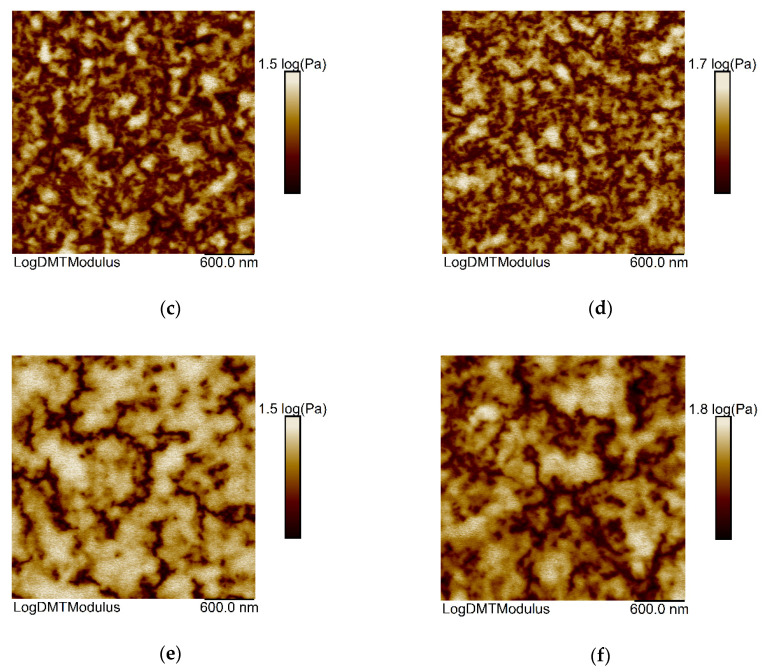
DMT-modulus scans by AFM of P1500 membranes: (**a**) Pristine membrane without additives; (**b**–**f**) Membranes with additives: (**b**) JED600PPG380; (**c**) JED600PPG640; (**d**) JED600TPT302; (**e**) JT403PPG640; (**f**) JT403TPT302.

Figure 5 presents images of TFCM surfaces obtained by AFM in the log DMT-modulus channel of the PF QNM mode. This channel represents the elasticity map according to the DMT model [41]. The bright parts of the map have a higher elasticity modulus than the darker regions. Three images per sample at separate spots reveal the fluctuation of the elasticity morphology within a sample (Figure S4), just like the histograms of these images (Figure S5). The corresponding height images give a rough impression of the domain size but lack a clear material contrast (Figure S6).

In the log, DMT images of the pristine P1500 sample, the regions of different brightness can be attributed to a fluctuation in the stiffness of PEG block (Figure 5a). This fluctuation of elasticity modulus could also be induced by the hard PBT block. However, as the portion of PBT (23 wt %) and therefore the length of this block in P1500 is roughly 2.2 moieties and, therefore, very small; it is not resolved in these AFM images and so not solely responsible for the stiffer phase. The comparison of the pristine membrane with TFCMs functionalized with additives unveils no distinguishable new phase after functionalization. Hence, the elasticity modulus of the additive matches the softer or stiffer phase, depending on the specific combination of amine- and glycidyl-containing additives. 

It is noteworthy that the composition JT403PPG640 induces a pronounced coarse distribution of softer and stiffer domains within the selective layer of the membrane, with respect to the pristine P1500 reference, while enhancing the stiffer portion (Figure 5e). The former observation is reflected by the largest domain size, the latter by the highest histogram peak ratio of stiff to soft phase (Table S4). On the contrary, the addition of JED600PPG640 or JED600TPT302 to P1500 leads to a finer local distribution of stiffness, enhancing the soft portion compared to the pristine TFCM (Figure 5c,d). Again, this observation is supported by the smallest domain sizes and smallest histogram peak ratios (Table S4). In addition, the JED600TPT302 additive causes a highly crystalline appearance, as visible in the height images (Figure S5), due to the high density of domain boundaries. These effects cannot be merely attributed to JED600, as JED600PPG380 produces a similar morphology and stiffness distribution to the pristine membrane. Equally, the different impact of JED600TPT302 and JED600PPG380, despite their epoxides’ similar molecular weight, like the absence of a trend in morphology from pristine via JED600PPG380 to JED600PPG640, rules out the chain length as a decisive factor. Moreover, the lack of a trend from di- to tri-epoxides emphasizes the absence of an effect regarding branching. In the case of membranes blended with additives having three reactive groups, there is a pronounced fine distribution of soft and stiff areas after the addition of JED600TPT302. On the contrary, the addition of JT403PPG640 does not change the distribution of the soft regions compared to the pristine TFCM. To explain these findings, we can only pinpoint the specific combination of additive components, whereas a single component of the additive seems not to be relevant for enlarged soft or hard regions.

**Figure 6:**
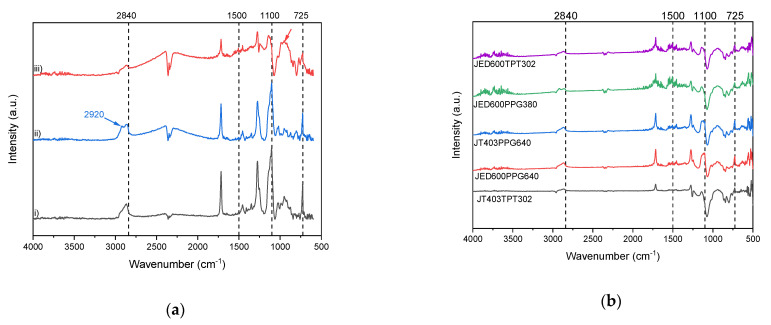
FTIR of coated on TFCM with gutter layer (**a**) pure P1500 (i); P1500 blended with nonreacted JED600 and PPG640 (ii); P1500 blended with reacted JED600 and PPG640 (iii). (**b**) P1500 blended with reacted additives: JT403TPT302; JED600PPG380; JT403PPG640; JED600PPG640; JED600TPT302.

In Figure 6a, the FTIR spectra of the selective layer of pure P1500 and P1500 blended with additives having two reactive groups with weight ratios 100/0 and 70/30 are shown. For all PEO-containing samples, a stretching signal appeared around 2800 cm^−1^. In the presence of glycidyl groups, samples blended with nonreacted additives revealed stretching at around 2920 cm^−1^, which disappeared after thermal treatment in the sample with reacted end-groups. A glycidyl‒amine reaction leads to the formation of a 3D network with the opening up of the oxirane ring and the formation of a secondary amine and ‒OH groups. For primary amines, the N‒H stretching is located from 1650 cm^−1^ to 1500 cm^−1^, while for secondary amines after thermal treatment it appears at 1580 cm^−1^ to 1490 cm^−1^ (Figure 6a (iii)). The quantification of ‒OH is quite difficult because of the overlapping of the band at around 3500 cm^−1^ [42].

Other compounds match the spectral features of the reacted JED600PPG640 sample, especially the absence of a pronounced glycidyl peak at 2920 cm^−1^ (Figure 6b). Thus, all chosen pairs of additives can react directly in the solid selective layer of the TFCM. For the additive JED600PPG380, some residual nonreacted glycidyl end-groups can provoke the appearance of an additional stretching peak around 2920 cm^−1^. Presumably, the reacted components for this sample do not form a dense network in the matrix of P1500, but can be located as uniformly distributed individual inclusions (Figure S3b). Since the successful reaction of the amine and glycidyl groups was indicated by FTIR and the modification of the morphology was revealed by AFM, we can confirm the formation of a penetrating network in the P1500 matrix composed of soft, low molecular components.

**Figure 7:**
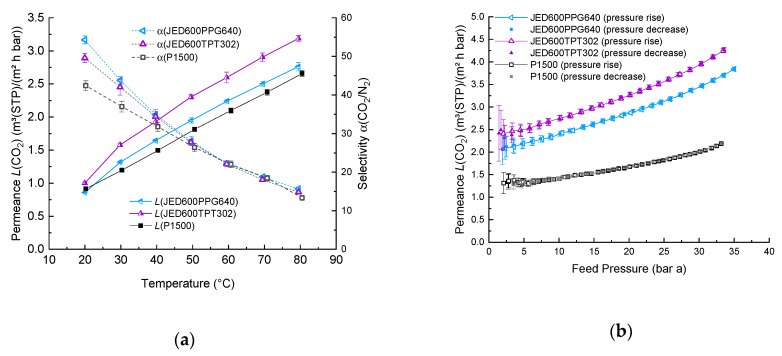
CO_2_ permeance and CO_2_/N_2_ selectivity of TFCMs with reacted additives (**a**) Low-pressure measurements in a wide temperature range. Error bars present instrumental uncertainties with a 95% level of confidence for 15 measurement points. (**b**) High-pressure measurements of TFCM with a selective layer composed of P1500 blended with reacted additives. Filled points are for a pressure rise measurement, unfilled for pressure decrease. Error bars present instrumental uncertainties.

Tailoring the design of TFCM with reacted blends for technical applications is a time-consuming task that entails additional costs. In order to control the reproducibility of results, the new samples with reacted additives JED600PPG640 and JED600TPT302 were prepared with a tailored thickness of 0.2 µm (Table S2). At temperatures above 30 °C, both JED600PPG640 and JED600TPT302 showed behavior that can be described by the Arrhenius equation (Figure 7a). The presence of reacted additives provokes earlier crystallization in the selective layer, causing higher selectivity at the same temperatures as compared to P1500. The effect of the improved selectivity in the region above 25 °C is related to the crystallinity shift for samples within the network [39].

High-pressure measurements from 2 to 35 bar and back to 2 bar were done for the same samples presented in Figure 7a in order to check the stability of TFCM with the reacted additives under a constant flow of CO_2_ through the membrane with a changing feed pressure. In the presence of both JED600PPG640 and JED600TPT302, an increase in CO_2_ permeance compared to pure P1500 was obtained in both low-pressure experiments carried out in the temperature range 10–80 °C (Figure 7a) and in the experiment with temperature fixed at 30 °C and feed pressure up to 35 bar. The conducted experiments clearly show that leaching out of the reacted additives does not take place at high temperature or at high pressure under continuous CO_2_ gas flow through the membrane. Membranes with networks formed of amine- and epoxy-terminated PEG and PPG additives in the P1500 matrix show superior gas separation properties compared to the pure P1500 TFCM.

## 4. Conclusions

The considered method of using reactive additives makes it possible to form networks directly in the polymer matrix of the separation layer of a TFCM. It is impossible to compare the formation and, correspondingly, the morphology of the embedded networks in a thick film with the formation in a thin-layer membrane, which is confirmed by the data of gas transport measurements.

The functional groups of the selected components affect the morphology and formation of embedded networks, as well as the transport properties. In thin films, a relatively small loss of selectivity is observed compared to thick films. This is especially true for the crystallization of additives in the polymer body. Thus, the choice of appropriate pairs of additives for the network formation proved an essential strategy for tuning the performance in a favorable direction.

In a thin layer of up to 200 to 300 nm, a network of additives is not formed in three dimensions; rather, they form separate sections in the region of the soft segment of the main polymer.

## Figures and Tables

**Table 1:** Properties of components used for the network formation in P1500 matrix.

Important Morphological Feature	Commercial Name	Code	Molecular Mass *M_W_*, (g/mol)	Density (at 25 °C) ^1^, (g/cm^3^)	Flash Point ^1^, (°C)
Matrix forming polymer	PolyActive^TM^ 1500PEGT77PBT23	P1500	1500 ^2^	1.188 ^3^	-
Two reactive glycidyl groups	Poly(propylene glycol) diglycidyl ether	PPG380	380	1.14	113
		PPG640	640	1.06	113
	Poly(ethylene glycol) diglycidyl ether	PEG526	526	1.14	197
Three reactive glycidyl groups	Trimethylolpropane triglycidyl ether	TPT302	302	1.157	n.d.
Two reactive amino groups	Jeffamine^®^ ED600	JED600	600	1.035	113
	Jeffamine^®^ ED900	JED900	900	1.065	113
	Jeffamine^®^ ED2003	JED2003	2000	1.068	113
Three reactive amino groups	Jeffamine^®^ T403	JT403	482	0.978	113

^1^ Specification sheet; ^2^ MW of PEGT [11]; ^3^ density measurements of polymeric film performed at HZG.

**Table 2:** HSP, total solubility and solubility distance parameters for CO_2_ and selected compounds at 25 °C and CO_2_ pressure of 1 bar.

Component	Molar Volume, (cm^3^/mol)	$\delta_{dp}$ (MPa^1/2^)	$\delta_{pp}$ (MPa^1/2^)	$\delta_{hp}$ (MPa^1/2^)	$\delta_{t},\;$ (MPa^1/2^)	Ra
Soft segment PEGT1500 ^1^	1249.4	18.8	11.9	9.4	24.2	9.94
Hard segment PBT ^1^	113.4	28.8	21.6	9.4	37.2	31.29
P1500 ^2^	n.a.	21.1	14.2	9.4	27.1	14.66
PPG380	370.4	13.9	7.8	7.6	17.7	4.6
PPG640	704.9	15.0	7.7	7.9	18.4	3.3
PEG526	281.9	16.0	10.0	8.7	20.8	5.65
TPT302	238.6	13.8	8.5	8.0	18.1	5.33
JED600	693.1	16.9	8.3	9.4	21.0	5.39
JED900	970.9	16.5	8.4	9.0	20.6	4.91
JED2003	1906.9	15.9	9.1	8.7	20.3	4.89
JT403	537.7	15.6	5.0	9.5	19.0	3.7

^1^ These components were observed as segments of P1500. ^2^ PolyActive^TM^ 1500 consists of 77 wt % PEGT and 23 wt % PBT.

**Table 3:** DSC measurements for selected components.

Component	T_g_, (°C)	T_c_, (°C)	T_m_, (°C)
P1500	−49	11 ^1^	28 ^1^
PPG380	−78	n.a.	n.a.
PPG640	−76	n.a.	n.a.
PEG526	−72	−45	−14
TPT302	−66	n.a.	n.a.
JED600	−49	−26	−12 (−10) ^2^
JED900	−64	2	21 (22) ^2^
JED2003	−56	−6	36 (43) ^2^
JT403	−69	n.a.	n.a.

^1^ From [37]; ^2^ Data from the producers are given in brackets.

**Table 4:** Permeability coefficients for various gases at 30 °C for blended thick film. The uncertainties estimation, with 95% confidence, is presented in parentheses.

Sample	P(O_2_), (Barrer)	P(N_2_), (Barrer)	P(CH_4_), (Barrer)	P(H_2_), (Barrer)	P(CO_2_), (Barrer)	S(CO_2_), (cm^3^(STP) cm^−3^ cmHg^−1^)	D(CO_2_), (10^−6^ cm^2^ s^−1^)
P1500 ^1^	8.7	3.4	10.5	17.6	181 (9.26)	0.019 (0.001)	0.91 (0.06)
Thick P1500 films blended with one component							
P1500-40%PPG380	20.0 (1.35)	7.9 (0.53)	27.3 (1.84)	34.4 (2.33)	368 (24.8)	0.025 (0.002)	1.504 (0.13)
P1500-40%PPG640	22.3 (1.9)	8.6 (0.73)	30.5 (2.59)	37.3 (3.17)	357 (30.3)	0.026 (0.002)	1.351 (0.16)
P1500-40%PEG526	15.5 (1.97)	6.4 (0.81)	21.1 (2.67)	26.7 (3.39)	326 (41.3)	0.024 (0.003)	1.349 (0.24)
P1500-40%TPT302	45.1 (4.49)	43.6 (4.20)	68.5 (6.34)	n.a.	246 (22.9)	0.026 (0.003)	0.963 (0.13)
P1500-40%JT403 ^2^	20.1 (1.2)	n.a.	n.a.	n.a.	3.5 (0.09)	0.581 (1.04)	0.0006 (0.011)
Thick P1500 films blended with two reactive components							
50%JED600PPG380	n.a	n.a.	n.a.	n.a.	208.9 (16.3)	0.034 0.004)	0.621 (0.008)
50%JED900PPG380 ^2^	n.a.	n.a.	7.9 (0.45)	n.a.	116.0 (6.70)	0.026 (0.001)	0.444 (0.036)
50%JED2003PPG380	11.2 (0.59)	4.1 (0.22)	14.3 (0.75)	22.3 (1.17)	212.8 (11.1)	0.024 (0.001)	0.003 (0.06)
50%JED600PEG526	7.2 (0.47)	4.9 (0.32)	8.6 (0.56)	32.0 (2.10)	96.7 (6.35)	0.136 (0.049)	0.072 (0.025)
50%JT403PPG380	3.0 (0.34)	n.a	2.4 (0.27)	12.0 (1.34)	22.8 (2.56)	0.180 (0.029)	0.018 (0.003)

^1^ Data published in [14]. ^2^ No data available for O_2_, N_2_ and CH_4_ due to insufficient film instability.

**Table 5:** Total solubility parameters for P1500 blend system.

Polymer Systems	$\delta_{dp}$ (MPa^1/2^)	$\delta_{pp}$ (MPa^1/2^)	$\delta_{hp}$ (MPa^1/2^)	*δ_t_*, (MPa^1/2^)	$R_{a}({\mathrm{CO}}_{2})$
P1500-40%PPG380	18.2	11.6	8.7	23.3	8.80
P1500-40%PPG640	18.6	11.6	8.7	23.6	9.28
P1500-40%PEG526	19.1	12.5	9.1	24.6	10.60
P1500-40%TPT302	18.2	11.9	8.8	23.5	9.03
P1500-40%JT403	18.9	10.5	9.4	23.6	9.24
50%JED600PPG380	18.2	11.1	8.9	23.2	8.55
50%JED900PPG380	18.2	11.1	8.9	23.1	8.42
50%JED2003PPG380	18.0	11.3	8.8	23.0	8.32
50%JED600PEG526	18.8	11.7	9.2	23.9	9.68
50%JT403PPG380	17.9	10.4	8.9	22.5	7.56
50%JT403TPT302	17.9	10.5	9.1	22.7	7.75

## Supplementary Materials

The following are available online at https://www.mdpi.com/2077-0375/11/3/174/s1, Table S1: Chemical structure of components used for network formation, Figure S1: Thin film composite membrane structure prepared for this study, Figure S2: DMT SEM images of upper surface of P1500 thick films: (a) Pristine film without additives; (b–f) Films with additives: (b) JED600PPG380; (c) JED600PPG640; (d) JED600TPT302; (e) JT403PPG640; (f) JT403TPT302; Table S2: Thicknesses of TFCM taken from SEM micrographs of the cross section. Standard deviations for 10 specimens are presented in parentheses, Figure S3: SEM images of upper surfaces of P1500 TFCMs: (a) Pristine membrane without additives; (b–f) Membranes with additives: (b) JED600PPG380; (c) JED600PPG640; (d) JED600TPT302; (e) JT403PPG640; (f) JT403TPT302. Table S3: DSC measurements for selected TFCM with network formation, Figure S4: DMT modulus scans of three spots on P1500 membranes: (a) Pristine membrane without additives; (b–f) Membranes with additives: (b) JED600PPG380; (c) JED600PPG640; (d) JED600TPT302; (e) JT403PPG640; (f) JT403TPT302, Figure S5. Histograms of the DMT-modulus scans of three spots on P1500 membranes: (a) Pristine membrane without additives; (b–f) Membrane with additives: (b) JED600PPG380; (c) JED600PPG640; (d) JED600TPT302; (e) JT403PPG640; (f) JT403TPT302. The black circle marks the frequency value used for normalization, Figure S6. AFM height scans of three spots on P1500 membranes: (a) Pristine membrane without additives; (b–f) Membranes with additives: (b) JED600PPG380; (c) JED600PPG640; (d) JED600TPT302; (e) JT403PPG640; (f) JT403TPT302, Table S4. Parameters of the stiffer phase’s lateral size (domain size by FWHM method) and proportion to softer phase (histogram peak ratio), averaged over the DMT images of three scans.
