# Supplementary material for: Multicomponent Network Formation in Selective Layer of Composite Membrane for CO2 Separation
Source: Membranes (Basel). 2021 Feb 28;11(3):174. doi: 10.3390/membranes11030174 (PMC7997254; doi:10.3390/membranes11030174)
Supplement: Supplementary file 1 [file membranes-11-00174-s001.zip › membranes-1047609_Supplementary/Lillepaerg_Supplementary information01_Multicomponent Network Formation.pdf]

membranes

Multicomponent Network Formation in Selective Layer of Composite Membrane for CO2 Separation

Jelena Lillepärg [1], Evgeni Sperling [1], Marit Blanke [1,2], Martin Held[1] and Sergey Shishatskiy [1]

[1] Helmholtz-Zentrum Geesthacht, Institute of Polymer Research, Max-Planck-Str. 1, 21502 Geesthacht, Germany; evgeni.sperling@hzg.de (E.S.); martin.held@hzg.de (M.H.); sergey.shishatskiy@hzg.de (S.Sh.)
[2] Current address: marit.blanke@gmx.de
• Correspondence: jelena.lillepaerg@hzg.de; Tel.: +49-4152-87-2448 (J.L.)

Supporting Information for the Manuscript

Table S1. Chemical structure of components used for network formation.

Commerzial name	Code	Chain structure	Fragment
PolyActive™	P1500	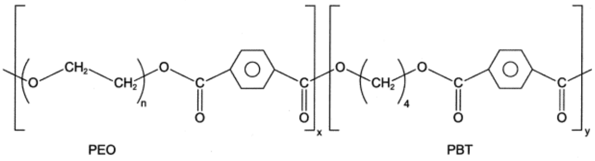	
Poly(propylene glycol) diglycidyl ether	PPG380<br>PPG340	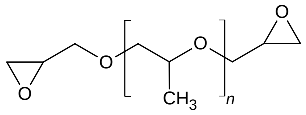	n~4.3<br>n~9
Poly(ethylene glycol) diglycidyl ether	PEG5265	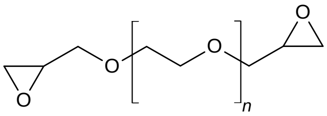	n~9
Trimethylolpropane triglycidyl ether	TPT302	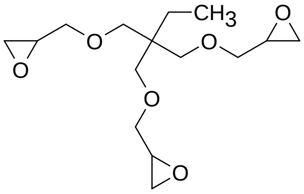	-
Jeffamine® ED-series	JED600<br>JED900<br>JED2000	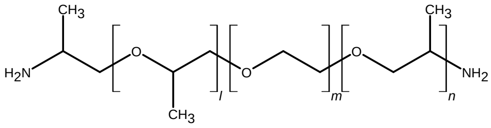	l~9.0; (m+n)~3.6<br>l~12.5; (m+n)~6.0<br>l~39; (m+n)~6.0
Jeffamine® T-series		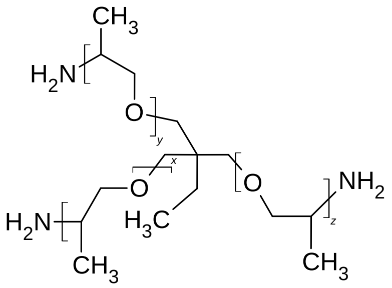	(x+y+z) ~ 5-6

Reaction between amino- and glycidyl-end groups:

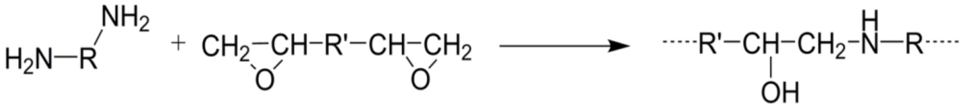

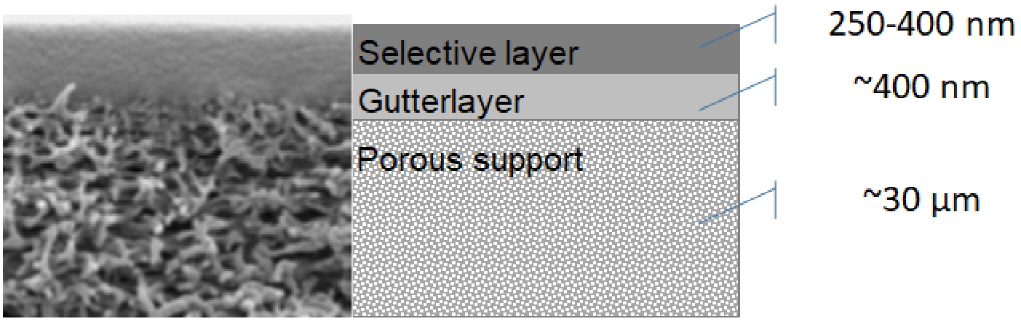

Figure S1. Thin film composite membrane structure prepared for this study.

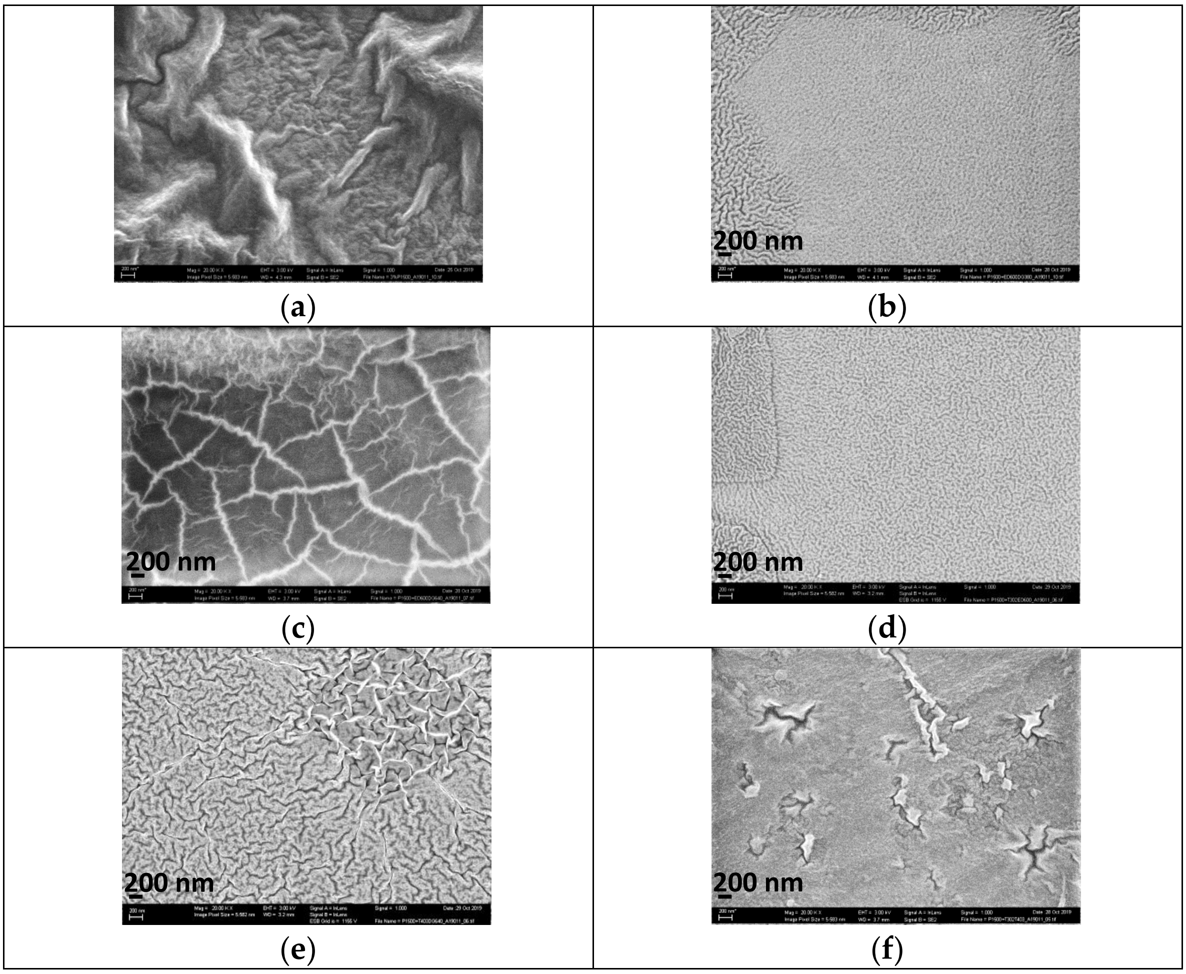

Figure S2. SEM images of upper surface of P1500 thick films: (**a**) Pristine film without additives; (**b**–**f**) Films with additives: (**b**) JED600PPG380; (**c**) JED600PPG640; (**d**) JED600TPT302; (**e**) JT403PPG640; (**f**) JT403TPT302.

Table S2. Thicknesses of TFCM taken from SEM micrographs of the cross section. Standard deviations for 10 specimens are presented in parentheses

Top layer of TFCM	Thickness of top layer (nm)
Gutter layer	226 (11)
P1500	214 (45)
P1500 with 30 % JED600TPT302 (Figure 3)[1]	315 (194)
P1500 with 30% JED600PPG640 (Figure 3)[1]	458 (91)

P1500 with 37 % JED600TPT302 (Figure 7)[2]	267 (70)
P1500 with 37 % JED600PPG640 (Figure 7)[2]	195 (69)

[1] Direct contact with polymers solution. [2] Using a subsidiary roll between solution bath and moving support gives the different in the thickness of coated layer.

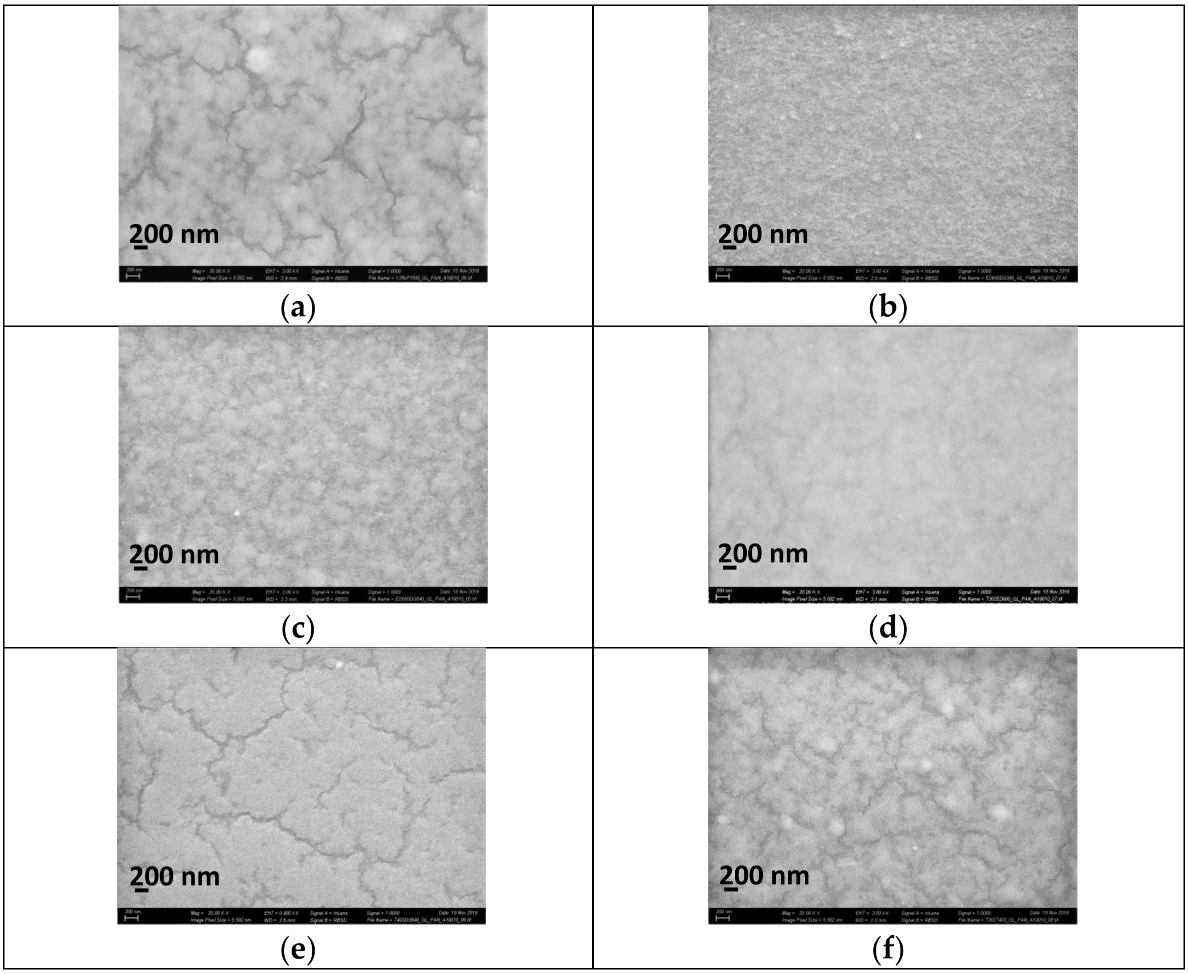

Figure S3. SEM images of upper surfaces of P1500 TFCMs: (**a**) Pristine membrane without additives; (**b**–**f**) Membranes with additives: (**b**) JED600PPG380; (**c**) JED600PPG640; (**d**) JED600TPT302; (**e**) JT403PPG640; (**f**) JT403TPT302.

Table S3. DSC measurements for selected TFCM with network formation.

**Component**	**T_g, (°C)**	**T_c, (°C)**	**T_m, (°C)**
P1500	-49	11[1]	28[1]
JED600PPG380	-30	5	28
JED600PPG640	-45	12	30
JT403TPT302	-47	5	28
JED600TPT302	-19	6	29
JT403PPG640	-25	10	30
