# Supplementary material for: Multicomponent Network Formation in Selective Layer of Composite Membrane for CO2 Separation
Source: Membranes (Basel). 2021 Feb 28;11(3):174. doi: 10.3390/membranes11030174 (PMC7997254; doi:10.3390/membranes11030174)
Supplement: Supplementary file 1 [file membranes-11-00174-s001.zip › membranes-1047609_Supplementary/Lillepaerg_Supplementary information02_Multicomponent Network Formation.pdf]

membranes

Multicomponent Network Formation in Selective Layer of Composite Membrane for CO2 Separation

Jelena Lillepärg [1], Evgeni Sperling [1], Marit Blanke [1,2], Martin Held[1] and Sergey Shishatskiy [1]

[1] Helmholtz-Zentrum Geesthacht, Institute of Polymer Research, Max-Planck-Str. 1, 21502 Geesthacht, Germany; evgeni.sperling@hzg.de (E.S.); martin.held@hzg.de (M.H.); sergey.shishatskiy@hzg.de (S.Sh.)
[2] Current address: marit.blanke@gmx.de
• Correspondence: jelena.lillepaerg@hzg.de; Tel.: +49-4152-87-2448 (J.L.)

Supporting Information for the Manuscript

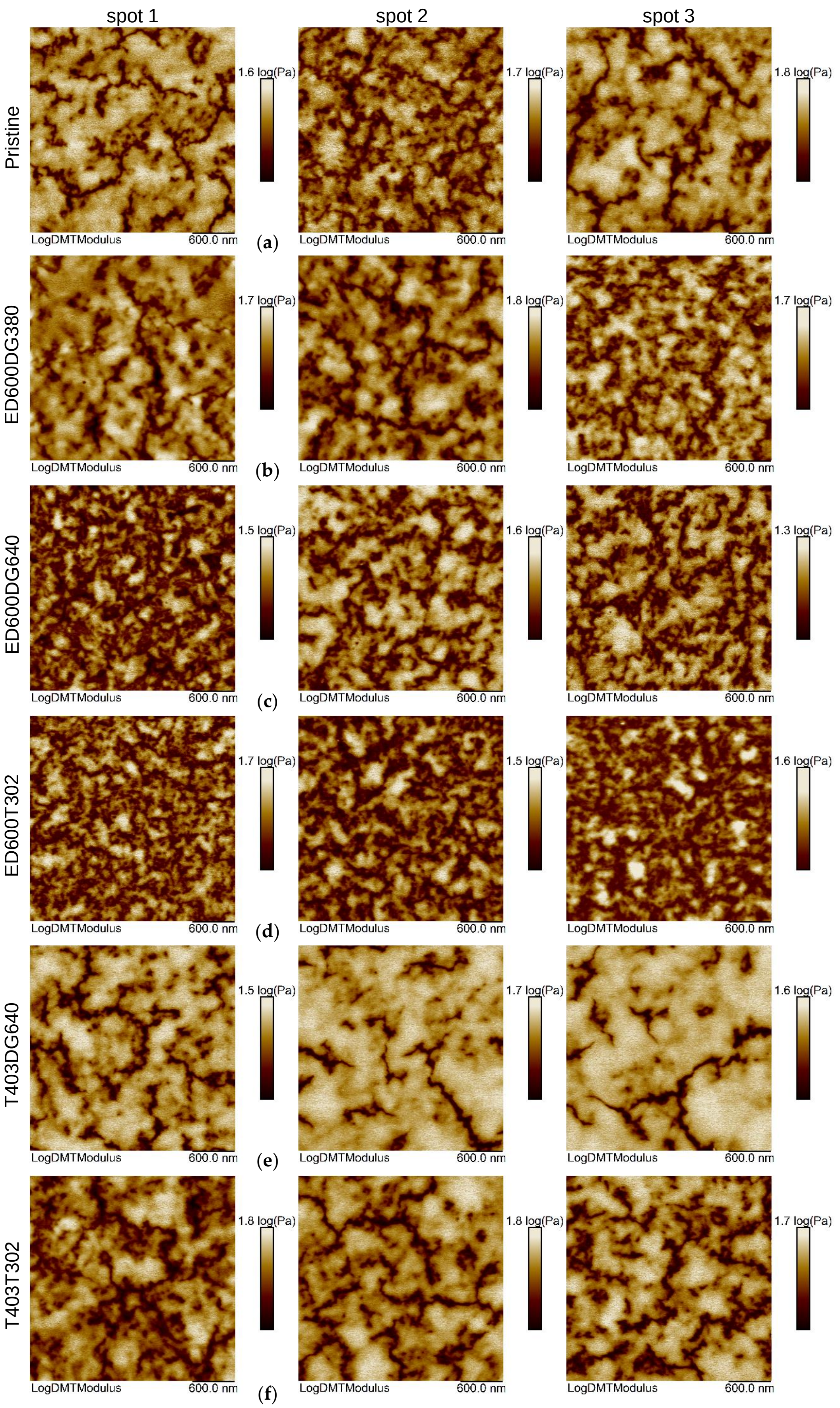

9 **Figure S4.** DMT-modulus scans of three spots on P1500 membranes: (**a**) Pristine membrane without
10 additives; (**b-f**) Membranes with additives: (**b**) JED600PPG380; (**c**) JED600PPG640;
11 (**d**) JED600TPT302; (**e**) JT403PPG640; (**f**) JT403TPT302.

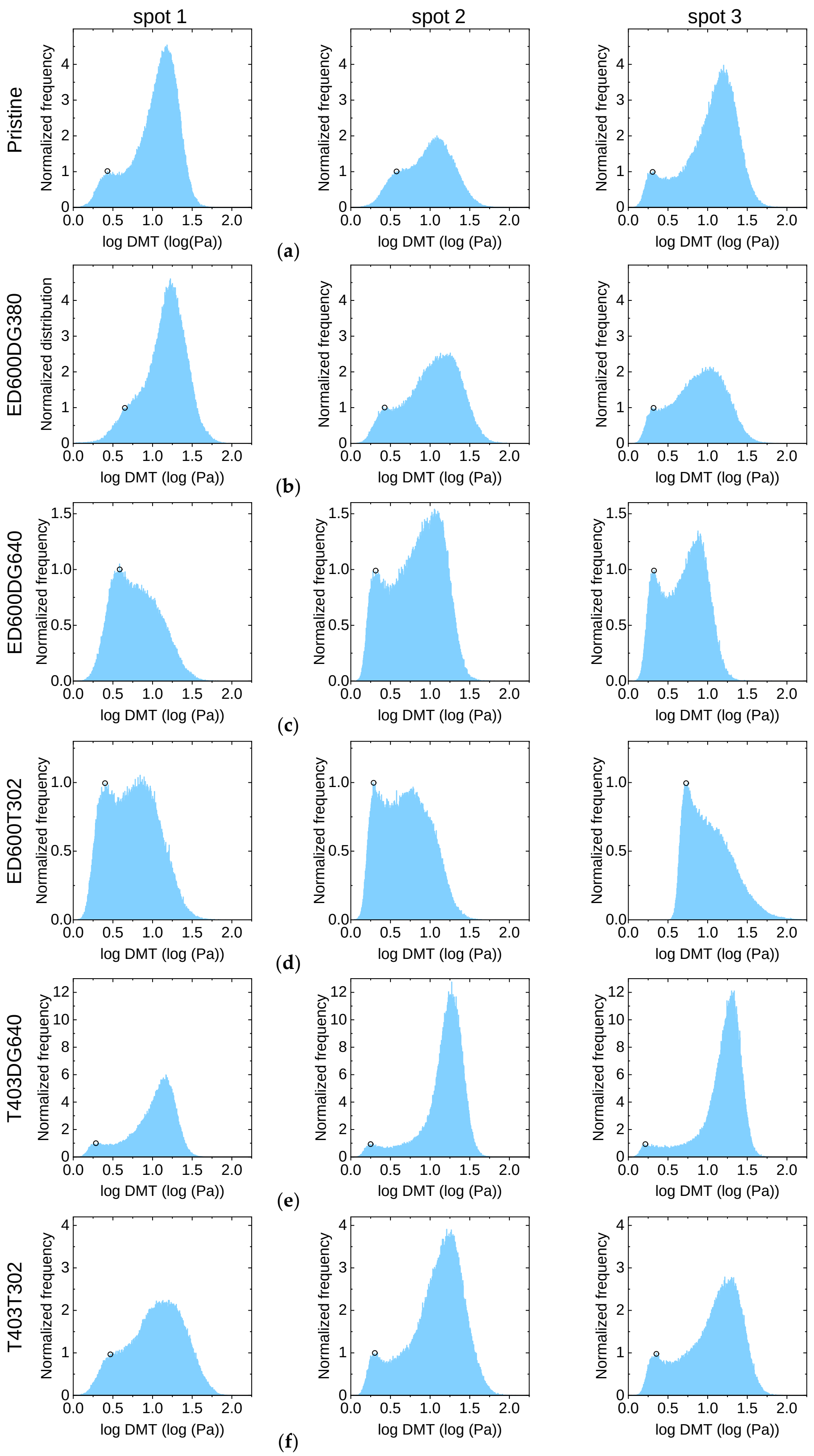

Figure S5. Histograms of the DMT-modulus scans of three spots on P1500 membranes: (**a**) Pristine membrane without additives; (**b-f**) Membrane with additives: (**b**) JED600PPG380; (**c**) JED600PPG640; (**d**) JED600TPT302; (**e**) JT403PPG640; (**f**) JT403TPT302. The black circle marks the frequency value used for normalization.

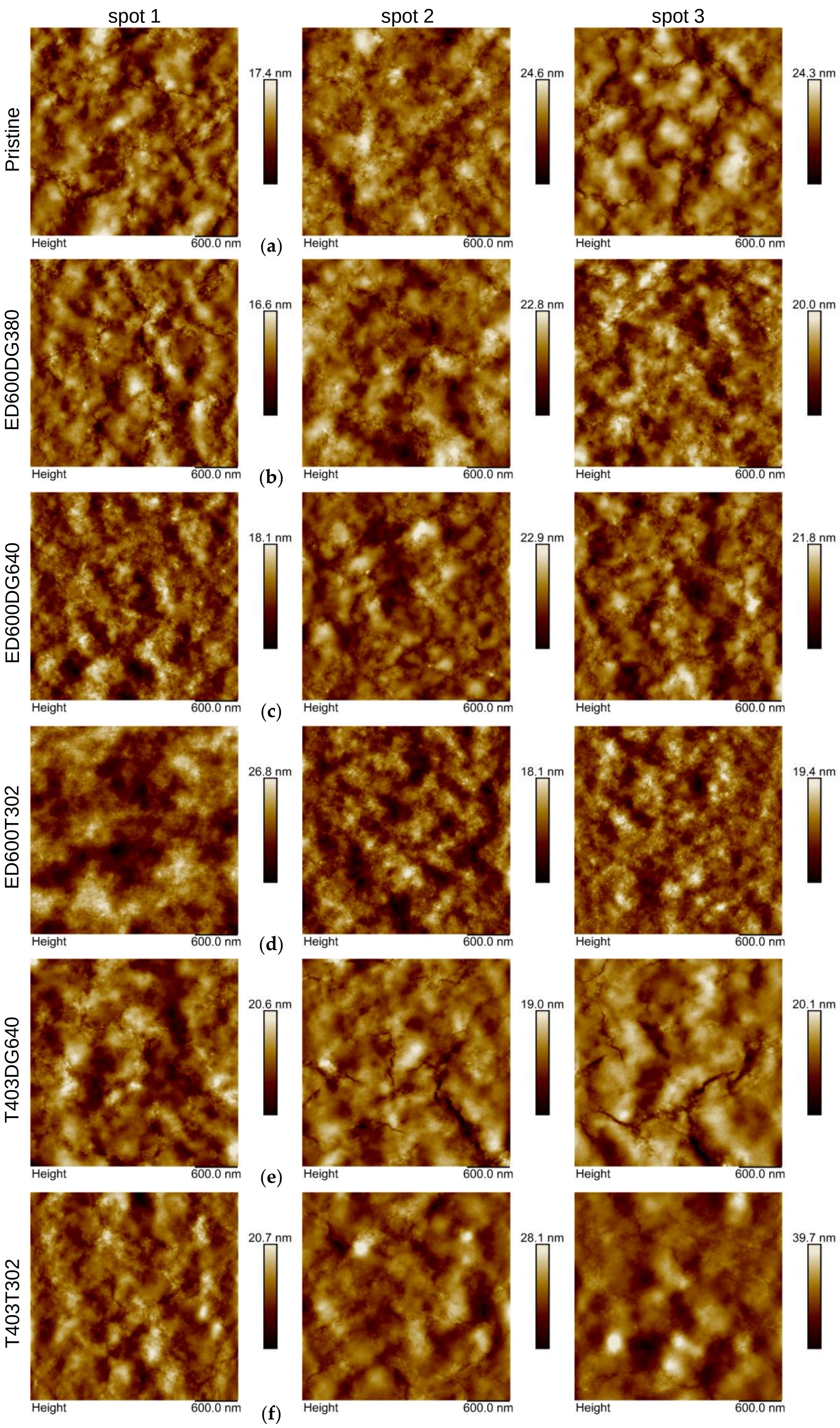

16 **Figure S6.** AFM height scans of three spots on P1500 membranes: (**a**) Pristine membrane without
17 additives; (**b-f**) Membranes with additives: (**b**) JED600PPG380; (**c**) JED600PPG640;
18 (**d**) JED600TPT302; (**e**) JT403PPG640; (**f**) JT403TPT302.

Table S4. Parameters of the stiffer phase's lateral size (domain size by FWHM method) and proportion to softer phase (histogram peak-ratio), averaged over the DMT images of three scans.

	**Pristine**	**JED600 PPG380**	**JED600 PPG640**	**JED600 TPT302**	**JT403 PPG640**	**JT403 TPT302**
Domain size (μm)	0.23 ± 0.21	0.26 ± 0.27	0.15 ± 0.13	0.11 ± 0.07	0.49 ± 0.48	0.3 ± 0.24
Histogram peak-ratio	3.4 ± 1.3	3.1 ± 1.3	1.2 ± 0.4	0.9 ± 0.2	9.9 ± 3.5	2.9 ± 0.8
